# Bacteriophages targeting intestinal epithelial cells: a potential novel form of immunotherapy

**DOI:** 10.1007/s00018-017-2715-6

**Published:** 2017-11-21

**Authors:** Andrzej Górski, Ewa Jończyk-Matysiak, Marzanna Łusiak-Szelachowska, Ryszard Międzybrodzki, Beata Weber-Dąbrowska, Jan Borysowski

**Affiliations:** 1 Bacteriophage Laboratory, Hirszfeld Institute of Immunology and Experimental Therapy, Polish Academy of Sciences (HIIET PAS), 53-114 Wrocław, Poland; 2 Department of Clinical Immunology, Transplantation Institute, Medical University of Warsaw, 02-006 Warsaw, Poland

**Keywords:** Phage, Intestinal epithelial cell, Immunity, Intestinal inflammatory disorder, Immunomodulation, Phage therapy

## Abstract

In addition to their established role as a physical barrier to invading pathogens and other harmful agents, intestinal epithelial cells (IEC) are actively involved in local immune reactions. In the past years, evidence has accumulated suggesting the role of IEC in the immunopathology of intestinal inflammatory disorders (IBD). Recent advances in research on bacteriophages strongly suggest that—in addition to their established antibacterial activity—they have immunomodulating properties that are potentially useful in the clinic. We suggest that these immunomodulating phage activities targeting IEC may open novel treatment perspectives in disorders of the alimentary tract, particularly IBD.

## Introduction

A central element of the intestinal barrier separating the body from the contents of the intestine is the IEC, playing a fundamental role in the absorption of nutrients and maintaining homeostasis [1]. IEC achieve this goal by working in concert with lymphoid, myeloid, and stromal cells. However, evidence has accumulated indicating that IEC not only act as a physical barrier to commensal bacteria and foreign antigens but are also actively involved in antigen processing and immune cell regulation [2].

## Bacteriophages as potential immunomodulators of IEC-dependent immunity

Bacterial viruses (bacteriophages, phages), which have the ability to multiply only in bacterial cells, are detectable where live bacteria exist and can be isolated from all environments (inland waters, hot springs, soil, foods, etc.). It is estimated that phages outnumber bacteria tenfold. Their selective action against bacteria and lack of harmful effects on eukaryotic cells has led to greatly increased interest in their potential in treating antibiotics-resistant infections [3]. Moreover, as first noted by the discoverer of phages, Felix d’Herelle, phages can be occasionally detected in blood and feces of animals and humans [4].

In 2005, we presented a hypothesis on the protective potential of such “endogenous” phages (“natural phage therapy”) present in feces, saliva, sputum, blood, urine, and other specimens. Based on our initial data, we also postulated that those endogenous phages (especially those abundantly present in the gut) may mediate immunomodulating properties contributing to maintenance of immunological homeostasis in the intestines [5]. Furthermore, we envisaged that phages can translocate from the gut and migrate to lymph, blood, and internal organs [6]. It has shown that phages were significantly enriched within the mucus surfaces as compared to the non-mucosal environment. Those phages adherent to mucus may reduce microbial colonization, thus providing a non-host derived immunity [7, 8], which essentially confirms our hypothesis. However, it should be highlighted that our vision extends the current understanding of phages and suggests their role not solely in antibacterial protection but also as immunomodulators contributing to maintenance of immunological homeostasis, particularly in the gut [5].

This vision has been recently supported by the concept of the “intrabody phageome” encompassing the collection of phages residing within different regions of the body [8]. Further work of our and other groups confirmed those assumptions, demonstrating that, indeed, phages may exert a variety of immunosuppressive functions in vitro and in vivo, causing extension of allograft survival and ameliorating symptoms of experimentally induced arthritis. Of particular interest are experimental, clinical, and laboratory data, indicating that phages can mediate anti-inflammatory effects which are independent of their well-established antibacterial effects [3, 9, 10]. Of particular importance is the recent work of Van Belleghem et al. [11]. Studying the effect of phages on human peripheral blood mononuclear cells, the authors found that phages downregulate the expression of CD14 and TLR4 (sensors of LPS whose activation induces secretion of proinflammatory chemokines and cytokines) as well as lysozyme. Importantly, phages upregulated IL-10 known for its potent anti-inflammatory properties.

It has been demonstrated that human IEC constitutively express an anti-inflammatory cytokine interleukin 1 receptor antagonist (IL1RN) contributing to mucosal protection which is upregulated by inflammation [12]. While Il1RN secretion by IEC of patients with IBD is slightly increased, this does not appear to counterbalance greatly increased production of proinflammatory cytokines [13]. Therefore, phage-dependent induction of IL1RN could dampen proinflammatory action of those cytokines [11].

Suppressor of cytokine signalling-3 (SOCS3) is a major regulator of inflammation [14]. Its overexpression may limit injury-induced epithelial hyperproliferation and inflammation-associated colon cancer [15]. BAFF molecule recently identified as being crucial for B lymphocyte functions is upregulated in IEC in inflammation and may contribute to the development of IBD. Increased expression of SOCS3 mediates the suppression of BAFF. There are suggestions that BAFF targeting may have therapeutic implications in IBD [16]. Of note, phages were shown to be upregulators of SOCS3 [11].

Oxidative stress has been suggested as a major contributing factor in the development of IBD: substantial evidence suggests that this syndrome represents a disturbed balance between increased reactive oxygen species (ROS) and decreased anti-oxidant activity. Anti-oxidant therapy was found to reduce disease activity in a mouse model of colitis [17]. However, clinical trials have not provided a conclusive answer; risks associated with compounds tested also inhibit further progress in this field [18]; phages are known to inhibit excessive ROS production [9], while phage therapy appears to be safe and relatively free of side effects [19].

Platelets (PLT) are important key regulators of inflammatory disorders beyond hemostasis and thrombosis [20]. IBD is associated with abnormalities in PLT function, whose aggregates may be present within mucosal microthrombi [21]. PLT seem to also be involved in amplifying inflammation-induced transendothelial leukocyte recruitment and activation [22]. In rats clapidogrel, a PLT activation suppressor resolved IBD symptoms [20]. Patients with IBD have increased risk for systemic thrombosis and no convincing data are yet available demonstrating that anti-PLT strategy used so far has been clinically useful, so the development of novel strategies targeting PLT is needed [23]. Phages can inhibit PLT adhesion to and PLT aggregation by fibrinogen in vitro [9].

**Fig. 1:**
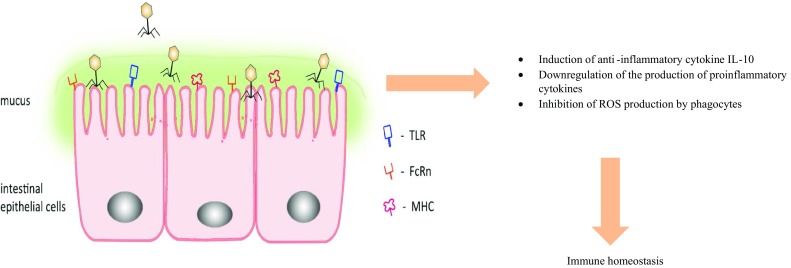
Phage interactions with IECs. Naturally occurring or administered phages interact with IEC and contribute to maintenance of immune homeostasis by inhibiting local aberrant immune and inflammatory reactions. FcRn (neonatal FC receptor)

Those data strongly support our concept of phage-mediated downregulation of aberrant immune response in the gut—associated lymphoid tissue [5, 9] but of course do not exclude that similar interactions could occur between phages and IEC (Fig. 1).

Data on phages in the gut of patients with intestinal diseases are scarce, so no clear association between their presence and disease can be currently established, although some data may suggest their protective role. For example, in some IBD (ulcerative colitis, Crohn’s disease), a common set of phages shared among healthy individuals (“healthy gut phageome”) was found to be markedly decreased [8, 24]. Likewise, reduced phage diversity was demonstrated in type I diabetes susceptible children (known to have an autoimmune background) [25]. Human stem cell transplant recipients who developed gastrointestinal graft versus host disease showed a reduction in the different types of gut phages, thus, suggesting that they may play a role in preventing this serious post-transplant complication [26]. Interestingly, in *Clostridium difficile* infection, phage transfer during fecal transplantation is associated with treatment outcome, even though there is no evidence that phages specific for that pathogen are being transferred [27, 28]. It is known that *C. difficile* causes colitis by inducing the expression of proinflammatory cytokines and cytotoxicity in colonic IEC in vitro and in vivo [29, 30]. Thus, the beneficial role of phages in this clinical setting may be associated primarily with their anti-inflammatory action at the level of IEC. Likewise, a phage cocktail against adherent invasive *Escherichia coli* abnormally predominant in the ileal mucosa of Crohn’s disease patients eliminated this pathogen from murine and human intestinal samples. What is more, this phage preparation strongly reduced dextran—induced colitis in mice colonized with this pathogen [31]. Furthermore, enteric viruses (where temperate phages predominate) ameliorate experimental gut inflammation in mice [32].

## IEC and immunity

IEC possess specialized receptors of the pathogen recognition receptor (PRR) family, such as Toll-like receptors (TLRs) and others, which recognize highly conserved molecular structures in microorganisms, leading to the activation of inflammation. Almost all TLRs are present in IEC, with differences in regard to their distribution along the intestinal tract [33]. IEC secrete and respond to cytokines. Importantly, IEC constitutively express and may secrete thymic stromal lymphopoietin (TSLP) which acts on dendritic cells with subsequent decreased production of proinflammatory cytokines IL-12 and IL-25 and increased production of IL-10. IL-10 is known for its ability to inhibit activation and effector function of T cells, monocytes, and macrophages. Therefore, its function appears to be to limit and ultimately terminate inflammatory responses [34] and mitigate immune pathologies [35]. IL-10 plays a major role in the maintenance of intestinal homeostasis; it is known that IL-10-deficient mice develop spontaneous colitis, while patients with a gene mutation in the IL-10 receptor develop severe IBD. It has been recently demonstrated that umbilical cord blood-derived mesenchymal cell extracts reduce experimental colitis in mice through their capacity to stimulate IL-10 production by macrophages [36]. Other recent data indicate that there exists, in the gut, a regulatory subpopulation of innate lymphoid cells which can be activated during inflammatory stimulation to secrete IL-10 favoring the resolution of intestinal inflammation [37]. In addition, IL-10 combined with IL-4 or IL-13 was shown to strongly suppress monocytic lysosomal enzyme release [38]. Those cytokines also downregulate MCP-1 production by activated IEC [39]. IEC also possess major histocompatibility complex: MHC-I and MHC-II molecules and peptides and can present antigens to T cells. Furthermore, IEC favour the translocation of intact antigens from the gut lumen [33]. Thus, it could be concluded that IEC maintain the balance between inflammation and tolerance. Main immunological functions of IEC are presented in Table 1.

**Table 1:** Main immunological functions of IEC

**Main cytokines produced by IEC**
Proinflammatory cytokines: TNF-α and IL-15 (in IBD patients) [2, 33]
Proinflammatory cytokines IL-1β and TNF-α causing an increase of MCP-1 production in vitro by IECs (chemokine playing role in intestinal inflammation in IBD) [2]
Anti-inflammatory cytokines IL-4, IL-10, IL-13 downregulating the production of MCP-1 in vitro by IECs and monocytic lysosomal enzyme release [39]
TGF-β causing suppression inflammation in neonatal gut [55]
**Main receptors**
TLRs (Toll-like receptors), which recognize microbe-associated molecular patterns and activate inflammatory mechanisms. TLRs have different expression in various parts of the intestine [2, 33]
MHC-I and MHC-II molecules responsible for antigen presentation to lymphocytes [33]
**Other functions**
The production of mucin proteins, i.e., TSLP^a^ which decreases the production of proinflammatory cytokines: IL-12 and IL-25 by DCs with simultaneously increasing production of IL-10 [33]
Influence the production of antibodies (sIgA) which prevent the adherence of antigens to gut mucosa [33]
Secretion of C3 complement component [40]
Production of serotonin [51]
Inhibition of PLT adhesion to and aggregation by fibrinogen [22]

References are provided in brackets

^a^TSLP (thymic stromal lymphopoietin)

As mentioned, IEC display class II antigens, a phenomenon dependent on IFN-gamma which upregulates their expression on those cells. Interestingly, abrogation of class II antigen expression on IEC may induce colitis in mice which suggests that INF-gamma exerts a critical anti-inflammatory function in the intestine which protects against colitis by inducing class II antigen expression on IEC. IEC constitutively produce and secrete the C3 component of complement. During intestinal inflammation, bacteria engage TLR4 on IEC which increases local C3 levels and the subsequent bacterial opsonization increases proinflammatory cytokine secretion [40].

Furthermore, it has been shown that the uptake and transportation ratios of nanoparticles by IEC in a state of inflammation are enhanced compared with the control [41]. This phenomenon has clear relevance for the uptake of phages by normal and inflamed IEC. Also, inflamed IEC can induce activation molecules on endothelial cells (ICAM-1, VCAM), a phenomenon that may lead to aggravation of inflammation. Interactions between IEC and endothelial cells may represent a mechanism for the gut epithelium to control the colonic inflammatory response and immune cell recruitment [42].

The intercellular communication of the immune system and IEC is bilateral: IEC secrete mediators (cytokines, etc.) acting on immune cells, while similar mediators produced by lymphocytes may regulate IEC functions, e.g., upregulating their MHC-II expression by IFN-gamma abundantly secreted by activated intraepithelial and lamina propria lymphocytes [43, 44].

Probiotic bacteria have been shown to mediate mainly anti-inflammatory responses in cultured IEC, whilst in vivo data in experimental animals are scarce [45–47]. They can also cause a TLR-dependent increase in the expression of IFN-alpha and beta in porcine IEC with subsequent improvement of the intestinal innate antiviral response and protection against intestinal viruses [47]. Furthermore, such anti-inflammatory bacteria also upregulate heat shock protein expression on colon IEC [48]. Recent data suggest that anti-inflammatory action of probiotics in the gut may also be mediated by IL-10 secreted by monocytes and macrophages [49].

Intestinal M cells are specialized IEC overlying lymphoid tissues in the small intestine; they may be induced by cytokines during inflammation in colonic epithelium and this process may be abrogated by anti-TNF-alpha blockade. M cells appear to be a correlate of chronic intestinal inflammation and a potential target for new treatment modalities of IBD [50]. Another type of specialized IEC is enterochromatin cells (EC), which constitute less than 1% of IEC and sense potentially noxious substances relaying information to the brain. EC produce more than 9% of body serotonin and are believed to affect a variety of pathophysiological states in the gastrointestinal tract. EC may be activated by products of commensals and transmit information to the nervous system [51].

## The involvement of IEC in IBD

Recently, significant advances have been made in understanding the interplay of the IEC, mucosal immune system, and microbiota in IBD [52–54]. Evidence has accumulated for aberrant IEC function in a variety of disorders of the intestinal tract, particularly inflammatory bowel diseases (IBD). An expansion of enterocytes producing IL-15 and TFN-alpha was observed in patients with IBD compared to healthy individuals. In addition, marked expression of IL-15 in the IEC of IBD patients has been confirmed by immunochemistry [55]. IL-15 is known to enhance immunogenicity through promoting the activation of dendritic cells [56] and is upregulated in leukocytes during sepsis [57]; therefore, targeting IL-15 within IEC may be a novel therapeutic option in patients with IBD. However, even though IL-6 is present in IEC of the small intestine and large intestine, no differences were found between IBD patients and controls [58].

It was shown that the epithelial cell layer of patients with Crohn’s disease is infiltrated by HLA-DR + T lymphocytes [59].

The role of epithelial TLR signalling in the pathogenesis of intestinal inflammation has been postulated. For example, activation of TLR4 in IEC causes inhibition of their migration and proliferation as well as the induction of apoptosis—factors promoting intestinal injury and inhibiting intestinal repair. Thus, IEC-specific TLR-based agents may be useful in the management of IBD [60, 61].

IEC of patients with IBD have been demonstrated to be deficient in their ability to normally stimulate suppressor cells [62]. In addition, IEC isolated from IBD patients have been shown to be hypersensitive to antigens derived from commensals, while those cells from healthy individuals were not, which suggests that immune tolerance of IEC to microbiota in IBD [63].

## Conclusion

The current advances in immunobiology of phages are in accord with the accumulation of new knowledge on the role of IEC in immunity. This parallel progress sheds new light on phages as factors contributing to maintenance of intestinal immune homeostasis, which is disturbed in some diseases of the gastrointestinal tract, particularly IBD. The initial experimental and clinical observations on intestinal phages support this notion of phage-dependent intestinal tolerance. This concept requires further study to broaden our understanding of phage immunobiology and its relevance in human pathology. In addition, it offers potentially new forms of phage-based therapies, since there is no approved agent targeting the epithelial barrier [64]. Importantly, progress in phage microencapsulation may enable their selective colon delivery [65].
